# Mapping the Spatial Distribution of Fibrillar Polymorphs in Human Brain Tissue

**DOI:** 10.3389/fnins.2022.909542

**Published:** 2022-06-01

**Authors:** Abdullah Al Bashit, Prakash Nepal, Theresa Connors, Derek H. Oakley, Bradley T. Hyman, Lin Yang, Lee Makowski

**Affiliations:** ^1^Department of Electrical and Computer Engineering, Northeastern University, Boston, MA, United States; ^2^Department of Bioengineering, Northeastern University, Boston, MA, United States; ^3^Massachusetts Alzheimer’s Disease Research Center, Boston, MA, United States; ^4^Department of Pathology, Massachusetts General Hospital, Boston, MA, United States; ^5^C.S. Kubik Laboratory for Neuropathology, Massachusetts General Hospital, Boston, MA, United States; ^6^Harvard Medical School, Boston, MA, United States; ^7^Department of Neurology, Massachusetts General Hospital, Boston, MA, United States; ^8^National Synchrotron Light Source II, Brookhaven National Laboratory, Upton, NY, United States; ^9^Department of Chemistry and Chemical Biology, Northeastern University, Boston, MA, United States

**Keywords:** Alzheimer’s disease, x-ray scattering, amyloid, plaques, neurofibrillary tangles, Aβ, tau

## Abstract

Alzheimer’s disease (AD) is a neurodegenerative disorder defined by the progressive formation and spread of fibrillar aggregates of Aβ peptide and tau protein. Polymorphic forms of these aggregates may contribute to disease in varying ways since different neuropathologies appear to be associated with different sets of fibrillar structures and follow distinct pathological trajectories that elicit characteristic clinical phenotypes. The molecular mechanisms underlying the spread of these aggregates in disease may include nucleation, replication, and migration all of which could vary with polymorphic form, stage of disease, and region of brain. Given the linkage between mechanisms of progression and distribution of polymorphs, mapping the distribution of fibrillar structures *in situ* has the potential to discriminate between mechanisms of progression. However, the means of carrying out this mapping are limited. Optical microscopy lacks the resolution to discriminate between polymorphs *in situ*, and higher resolution tools such as ssNMR and cryoEM require the isolation of fibrils from tissue, destroying relevant spatial information. Here, we demonstrate the use of scanning x-ray microdiffraction (XMD) to map the locations of fibrillar polymorphs of Aβ peptides and tau protein in histological thin sections of human brain tissue. Coordinated examination of serial sections by immunohistochemistry was used to aid in the interpretation of scattering patterns and to put the observations in a broader anatomical context. Scattering from lesions in tissue shown to be rich in Aβ fibrils by immunohistochemistry exhibited scattering patterns with a prototypical 4.7 Å cross-β peak, and overall intensity distribution that compared well with that predicted from high resolution structures. Scattering from lesions in tissue with extensive tau pathology also exhibited a 4.7 Å cross-β peak but with intensity distributions that were distinct from those seen in Aβ-rich regions. In summary, these observations demonstrate that XMD is a rich source of information on the distribution of fibrillar polymorphs in diseased human brain tissue. When used in coordination with neuropathological examination it has the potential to provide novel insights into the molecular mechanisms underlying disease.

## Introduction

Neurodegeneration in Alzheimer’s disease (AD) is accompanied by the progressive aggregation of Aβ peptides into plaques and tau proteins into neurofibrillary tangles. Spread and replication of tau aggregates follow well-established connections through the brain to generate characteristic patterns of location and abundance (Braak and Braak, 1991). Beta-amyloid (Aβ) plaque pathology also progresses in phases, following a general pattern somewhat different from that followed by tau (Thal et al., 2002). AD sub-types and other neurodegenerative diseases exhibit distinct trajectories of neurodegeneration (Murray et al., 2011), impact different regions of the brain and consequentially exhibit differences in clinical presentation. Each of these neuropathologies appears to be associated with a different set of fibrillar polymorphs that make up plaques (e.g., Cohen et al., 2015; Qiang et al., 2017; Di Fede et al., 2018) and/or tangles (Shi et al., 2021), suggesting a linkage between fibrillar structure and the trajectory of disease progression (Dujardin et al., 2020; Lau et al., 2021). How differences in disease phenotypes may be associated with differences in the structures of fibrils is unknown. While high resolution images of these pathological fibrillar structures have been determined, the structures of isolated fibrils have provided little insight into the way they contribute to disease progression. All known neuropathological fibrils have a highly stable cross-β core structure (e.g., Sunde et al., 1997). But they differ in protein or peptide composition; isoform; post-translational modifications (PTMs); conformational arrangement; and/or structural polymorph. Their varying composition, width, helical pitch, number of protofilaments and cross-sectional shape may all have implications for biological activity. We know what these structures look like, but we do not understand how they contribute to disease.

Progression of neuropathology during disease may involve nucleation, replication, and spreading of these pathological fibrils (Meisl et al., 2021). If fibrils spread in a prion-like manner—in which replication-competent seeds migrate through tissue and replicate to form identical copies of themselves—one might expect the structures of all pathological fibrils in a subject to be identical. However, if spread of fibrils also includes independent nucleation events, a diversity of polymorphs may be generated within the same subject. Mapping the molecular structures of fibrils *in situ* may make it possible to distinguish between these two mechanisms. Unfortunately, most methods capable of distinguishing between conformational polymorphs, such as cryoEM or ssNMR, require isolation of fibrils and consequent destruction of positional information. Cryo electron tomography can provide images of aggregates and their interactions with cellular components in micron-thick sections, but lacks the resolution to distinguish among polymorphs (Fitzpatrick and Saibil, 2019). Optical mapping can provide partial information through the use of conformation-specific antibodies or a panel of fluorescent dyes that exhibit distinct fibrillar binding affinities (Condello et al., 2018). However, what is needed is a method for generating structural information about polymorphs *in situ* at much higher resolution than optical methods. In this paper, we demonstrate that scanning x-ray microdiffraction (XMD) has the potential to produce this kind of information.

XMD involves the use of x-ray microbeams (5 μ diameter or smaller) to query the molecular structures present in unstained histological thin sections of human brain tissue. With the introduction of micron-sized x-ray beams for structural analysis (Yang et al., 2022), advanced synchrotron x-ray sources and high data rate detectors, the rapid scanning of millimeter-sized regions of thin sections of tissue has become feasible (Liu and Makowski, 2022). Until recently, X-ray scattering from thin sections of tissue was rarely attempted because the heterogeneous mixture of constituents makes data interpretation difficult, the disruption of native structure intrinsic to sample preparation may destroy relevant structural features and the disorientation and disorder of structures that do survive weaken and obscure the collected data. However, the development of micro- and nano-beams has made possible measurement of scattering from very small volumes which, in some cases, may be populated largely by a single constituent. Furthermore, pathological fibrillar protein deposits implicated in Alzheimer’s disease and other neurodegenerative diseases have robust architectures that are particularly resilient to the physical and chemical insults of sample preparation and radiation damage due to incident x-rays. Although fixed or frozen tissue can be used, here we describe experiments utilizing fixed tissue. Formaldehyde fixation and ethanol dehydration create a rigid cross-linked polymer matrix. Extensive studies on the chemistry of formaldehyde reaction with amino acids and proteins (French and Edsall, 1945; Werner et al., 2000) have shown that most protein secondary structures (Zohdi et al., 2015) and epitopes (Werner et al., 2000) are preserved in fixed tissue sections. Ethanol dehydration removes most lipids (Zohdi et al., 2015). Amyloid fibrils stabilized by a core of cross-β structure appear to be highly resilient to these treatments and are left largely intact, trapped within the cross-linked tissue matrix (Liu et al., 2016). Consequently, use of XMD for the study of fixed tissue sections from Alzheimer’s subjects should provide information on the conformation, organization and distribution of pathological fibrils *in situ*.

XMD cannot be used to directly generate high-resolution images of fibrils. The complex mixture of constituents, disorder, and disorientation preclude that. However, it can be used to distinguish between polymorphs, the structures of which have been determined *in vitro* or *ex vivo* by high-resolution techniques. Information from XMD can be classified into that associated with wide-angle scattering (WAXS) and with small-angle scattering (SAXS), which are collected simultaneously during an experiment. Wide-angle scattering includes the prototypical 4.7 Å reflection that is a consequence of the 4.7 Å periodicity of β-strands arranged parallel to one another, but perpendicular to the long-axis of a fibril in cross-β structures. The β-strands literally act like a diffraction grating to reflect x-rays at a characteristic angle, readily observed in the WAXS portion of the pattern. The scattered intensities in the WAXS portion of the pattern are highly sensitive to the fold of the proteins or peptides making up the fibril and the arrangement of protofilaments within the fibril, making it possible to distinguish between polymorphs on the basis of the shape of the 4.7 Å and other wide-angle reflections. Small-angle scattering provides information on the overall diameter and shape of the fibrils as well as the interactions they make with the surrounding environment (Roig–Solvas and Makowski, 2017), providing a measure of the density and organization of the microenvironment of the lesions in which they are embedded. Information on larger scale distribution and organization of fibrillar structures derives from the coordination of XMD with detailed neuropathological examination of serial sections which can confirm the molecular identity of the constituents of a lesion, aiding in the interpretation of x-ray data and providing a larger-scale framework for correlating x-ray observations in different parts of a tissue and, ultimately, different regions of a brain.

Here we demonstrate the feasibility of this approach, using XMD with immunohistochemistry to identify structural variations in fibrillar structure *in situ*.

## Materials and Methods

### Sample Preparation

Samples were formalin fixed, prepared using a modification of standard neuropathological processes to produce 20 μ thick sections as previously described (Liu et al., 2016). These unstained sections are thicker than conventional histological sections in order to increase the volume of material irradiated and improve the signal to noise ratio of the scattering patterns collected. Tissue sections were mounted on either 1 × 1 cm^2^ mica films (12 μ thick) or 2.7 × 2.7 mm^2^ SiN membranes (1 μ thick) and then affixed to sample holders printed to LiX beam line specifications (see below). These holders were mounted directly on the LiX beam line stage (Yang et al., 2020).

### X-Ray Microdiffraction Data Collection

**FIGURE 1:**
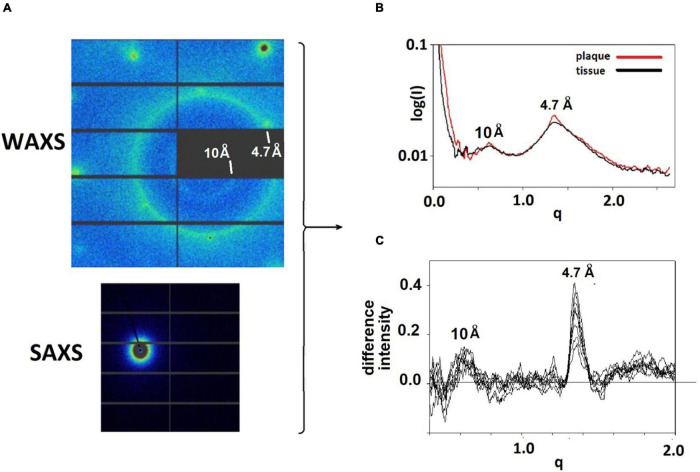
**(A)** WAXS and SAXS detector images from a pattern taken at the LiX beam line. Relatively sharp reflections at the left and top of the WAXS pattern are from the mica substrate. The strong circle of intensity is at 4.7 Å spacing, the weaker, smaller circle of intensity is at 10 Å spacing. The intensity seen in the SAXS detector is at very small angles and is the most intense in the pattern. **(B)** The intensities from the SAXS and WAXS detectors are circularly averaged and merged, resulting in scattering patterns that have similar characteristics at most positions in the sample. Regions with a high concentration of fibrillar structures exhibit sharp features at 4.7 and 10 Å spacing. These features can be seen in **(C)** plots of the difference between scattering from a lesion and scattering from tissue.

Tissue sections mounted on mica or SiN films were scanned with a 5 μ x-ray microbeam using 5 μ steps to collect diffraction patterns as a function of position on a square grid across the tissue section. Data collection was carried out at the LiX beam line at the NSLS-II synchrotron source at Brookhaven National Laboratory. Each region of interest (ROI) scanned constituted a rectangular area of between 300 × 300 μ^2^ (3,600 diffraction patterns) and 600 × 600 μ^2^ (14,400 diffraction patterns). An exposure time of 0.5 s was used and (including data transfer and sample step) approximately 0.8 s was required per exposure, or about 48 min for a 300 × 300 μ^2^ ROI. Scanning XMD generates two types of data—wide-angle (WAXS) that can be used to map the locations of specific polymorphs across multiple length scales; and small-angle (SAXS) that can be used to assess the molecular order within the microenvironment of lesions. Data were collected on SAXS and WAXS detectors simultaneously (see Figure 1), circularly averaged and merged using LiX-specific software.

### Data Processing

Intensities in diffraction patterns were pre-processed to remove scattering from the mica films (or SiN) used as substrates for the tissue. This involved the “masking” of peaks due to mica using a modification of LiX-specific software (Yang et al., 2020). Three of these peaks can be seen near the left and top edges of the diffraction pattern in Figure 1A. After masking, data from the SAXS and WAXS detectors were circularly averaged, merged and scaled, again using LiX-specific software, resulting in intensity distributions such as those in Figure 1B. The location of pathological protein deposits could usually be determined on the basis of intensity observed at q∼1.34 Å^–1^ (corresponding to a spacing of 4.7 Å) which is enhanced in scattering from the lesions. In the figures, scattering angle is approximated by the momentum transfer, q, which is defined as q = 4psin(θ)/λ where q is half the scattering angle and λ is the wavelength of the incident x-rays. Scattering from lesions is a mixture of scattering from fibrils and from tissue. The scattering from tissue within the lesion is often indistinguishable from that seen in proximal positions devoid of fibrils. This tissue scattering can be subtracted from the lesion scattering, resulting in estimation of scatter from fibrils as seen in Figure 1C for 9 patterns within a single lesion.

### Background Subtraction

Background scattering from surrounding tissue is removed from plaque scattering assuming that the structure of the tissue within a plaque is the same as that in a proximal region devoid of fibrils. For each pattern identified as potentially due to a lesion we scanned a 7 × 7 (35 ×35 μ) neighborhood centered on the pattern and chose the three patterns within that neighborhood exhibiting the lowest intensity in the q-range of 1.0–1.47 Å^–1^. In most cases, the scattering from adjacent tissue has an intensity distribution very similar to that of a lesion, but lower in overall intensity and absent a sharp peak at 4.7 Å spacing. This appears to be due to higher density of material within the thin section To compensate for this, tissue scattering intensity generally had to be scaled prior to subtracting it from plaque intensity. Since scattering from the underlying mica substrate does not require scaling, we calculated scattering from fibrils, I_f_, as: I_f_ = (I_p_–I_b_) –a (I_t_–I_b_), where I_p_ is the observed scattering from plaque; I_t_ that observed from proximal tissue; I_*b*_ background observed from scattering from mica devoid of tissue. The scale factor, a, was chosen to minimize the difference between plaque and tissue intensities in the range 1.6 < q < 2.0 Å^–1^, assuming that fibrils scatter very weakly in that region—an assumption consistent with all our observations to date.

### Calculation of X-Ray Scattering From Atomic Coordinates

**FIGURE 2:**
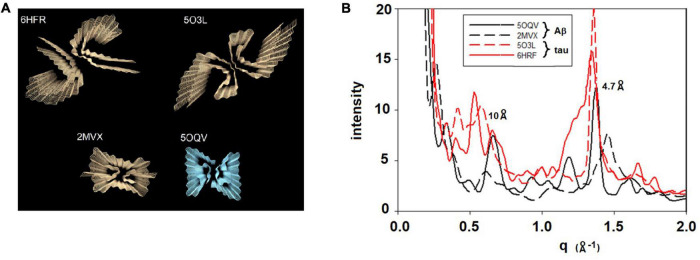
**(A)** Rendering of four cross-β fibrillar structures—top two are tau structures (from PDB files 6HFR and 5O3L) and bottom two are Aβ structures (from PDB files 2MVX and 5OQV). **(B)** Scattering patterns predicted from these four structures exhibit sufficiently large differences among them that they should be distinguishable on the basis of XMD data.

Since all fibrils of interest have a cross-β configuration, the question immediately arose as to whether scattering from different polymorphs is sufficiently different to be detected by XMD. To address this question, we calculated x-ray scattering from the atomic coordinates of fibrils imaged at high-resolution by cryoEM. For these calculations we used the program CRYSOL (Svergun et al., 1995), which was developed for predicting x-ray solution scattering from atomic coordinates. Scattering from particles embedded in a histological thin section is analogous to scattering from particles in aqueous solution. In both cases the particles are largely disoriented. The replacement of an aqueous environment with a complex polymeric matrix has strong impact on the SAXS portion of the scattering pattern and analysis of that impact will be reported elsewhere (Nepal et al., in preparation). Scattering in the WAXS portion of the pattern is not, however, strongly influenced by the microenvironment of the particles because it is due largely to intramolecular interference. The randomly placed polymers surrounding the particles contribute a diffuse background but do not influence the scattering from the fibrils. Atomic coordinates for dozens of amyloid structures are available in the Protein Data Bank with more being added every week. Generally, the deposited coordinates correspond to one or a few 4.7 Å—thick layers of a fibril. To generate the atomic coordinates for a long fibril, the coordinates of one layer were processed by a Matlab program that translated the coordinates of one layer by a multiple of 4.7 Å along the fibril axis and rotated the coordinates by an angle that was varied from –3*^o^* to + 3^o^ per layer. Typically, coordinates for a 30 layer (141 Å long) fibril were generated. Figure 2 exhibits the predicted scattering for two polymorphs each of Aβ and tau fibrils. As expected (Sunde et al., 1997), all fibrils are predicted to have a prominent peak at or near 4.7 Å spacing, but the shape of the peak, the intensity of the 10 Å peak, and the presence or absence of additional peaks is widely variable among fibrils. Differences of this magnitude are observable in WAXS data as described below. Both length and twist of a fibril have observable impact on the predicted scattering, resulting in a large library of potential structures and a corresponding library of predicted scattering patterns. Here we demonstrate that XMD data is of sufficient quality to distinguish among some, but not necessarily all, predicted patterns.

## Results

### Scattering From Histological Thin Sections

Figure 1 displays detector images from WAXS and SAXS detectors collected at the LiX beamline. WAXS data are collected on 9 detector chips as seen in the upper left of the Figure. Round, intense peaks near the edges of the detector are from the mica substrate on which the tissue was mounted The bright circle of intensity is at a spacing of 4.7 Å. The weak, smaller circle of intensity corresponds to the 10 Å scattering, also expected from a cross-β structure. Circular averaging and merging of SAXS and WAXS data generate a one-dimensional plot of intensity as a function of distance from the center of the pattern. Scattering from thin sections of fixed tissue generally exhibit *broad* peaks centered at ∼ 10 Å and 4.7 Å spacing, reflecting the partially denatured form of protein in the tissue debris within the section. Scattering from fibrillar structures produce a *sharp* feature at 4.7 Å scattering, generally located near the top of the broad peak from the tissue in which the fibrils are embedded.

Scanning XMD data was collected on 5 μ grids using a 5 μ diameter x-ray beam. Scans of several thousand diffraction patterns were collected and the resultant data sets were displayed as two-dimensional “heat maps” that were used to locate regions exhibiting strong intensity at 4.7 Å spacing, likely to highlight the locations of lesions containing cross-β structures.

### Mapping Locations of Fibrils in Tissue

**FIGURE 3:**
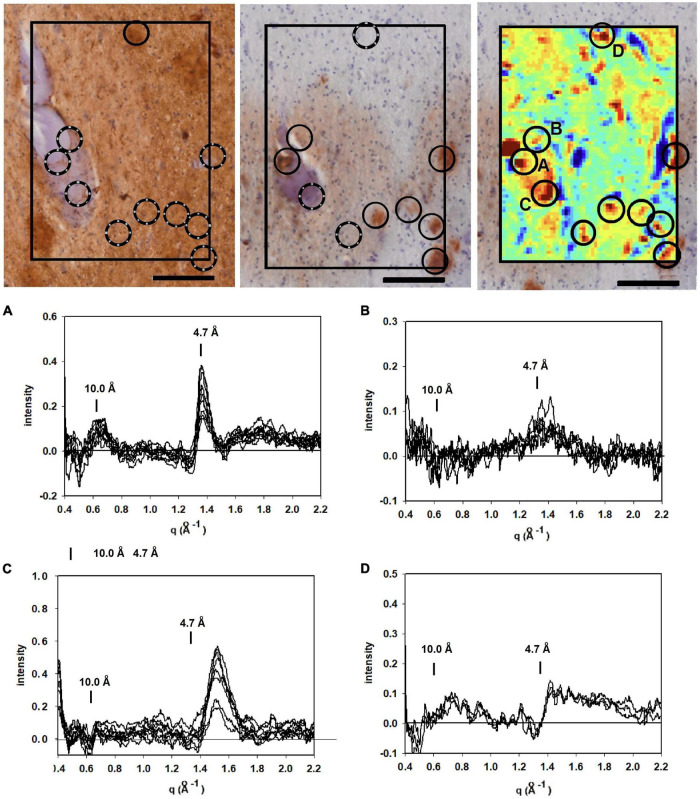
Images of serial sections of amygdala immunostained for tau (left); and Aβ (middle) compared with (right) a mapping of the 4.7 Å intensity observed from an unstained serial section (sequence of sections collected were tau→unstained→Aβ). Selected common features are marked by circles. Lesions seen in the heat map but missing in the stained section are indicated by broken circles. **(A–D)** WAXS scattering intensity from multiple patterns collected from each of four of the prominent features (features **A–D** in the x-ray map) are exhibited below. Scale bar 100 μ).

Figure 3 (middle) is a section immunostained for Aβ taken from the amygdala of a 53 year old woman with a family history of Alzheimer’s disease. Two members of her family were found to have a presenilin mutation and, on autopsy, exhibited histological features essentially identical to this subject. Neuropathology reported innumerable “cotton wool plaques” and numerous large, ill-defined amyloid plaques, consistent with Thal stage 5. Frequency and distribution of neurofibrillary tangles were consistent with Braak and Braak stage VI. Immunohistochemistry indicated both tau (left-hand panel) and Aβ (middle panel) were present in the amygdala of this subject as seen in the micrographs in Figure 3.

To supplement the information from neuropathology, we carried out scanning XMD on several samples from this subject including the 300 × 400 μ scan of 4,941 diffraction patterns (81 × 61) shown in the right hand panel in Figure 3. The distribution of intensity at 4.7 Å spacing was displayed as a “heat map” and compared with the adjacent serial sections stained for tau and Aβ. At least 7 features in the heat map correspond with stained lesions that appear in the micrograph of the serial section immuno-stained for Aβ; one feature in the heat map corresponds to a tau-staining lesion as marked by circles. Not all features in the heat map correspond to lesions observed by immunohistochemistry. Similarly, not all lesions observed by immunohistochemistry have corresponding features in the heat map. This is not surprising since these are images of three adjacent, 20 μ thick serial sections—not the same section—and features prominent in one section may not extend into the next section. Furthermore, the attributes visualized in the two figures are derived from different imaging modalities and reflect different properties of the tissue.

**FIGURE 4:**
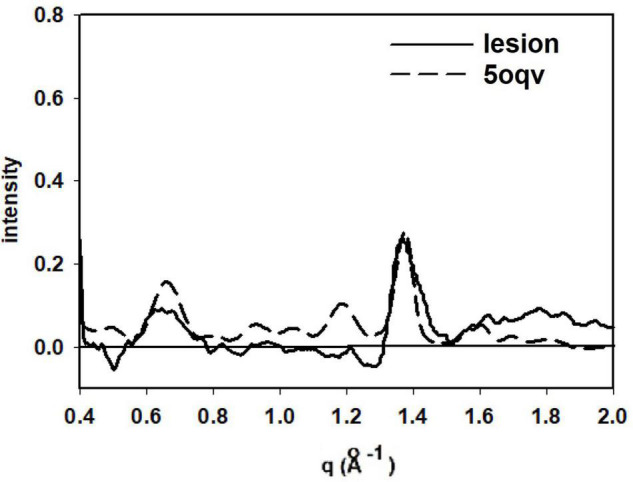
Comparison of the scattering from lesion A (in Figure 3) with that predicted from the atomic coordinates in PDB file 5oqv. The patterns share prominent features at q = 0.6 Å^–1^ (10 Å spacing), 1.34 Å^–1^ (4.7 Å spacing) and weaker features at q ∼ 0.9 Å^–1^ and 1.2 Å^–1^. strongly suggesting that the fibrils in lesion A have a structure very similar to that in the *in vitro* assembled Ab42 fibrils described by PDB file 5oqv.

Detailed analysis of the scattering from lesions identified in this heat map provide molecular-level information about the constituents. Tissue scattering was subtracted from the patterns arising from lesions A, B, C and D in the heat map and the resultant difference intensities are displayed in Figure 3 (bottom). Lesion (A) has a strong, sharp peak at 4.7 Å, indicative of a high concentration of cross-β structures. A prominent peak at q ∼ 0.6 Å^–1^ is also observed. This corresponds to a spacing of 10 Å, the distance between β-sheets that stack face-to-face. This scattering is prototypical of cross-β fibrils and, as seen in Figure 4, compares well to that predicted from the atomic coordinates of an *in vitro* assembled Aβ42 fibril as visualized by cryoEM (pdb file 5oqv—Gremer et al., 2017). X-ray scattering from lesion B, has a distinctly different intensity distribution which may indicate that the lesion is populated largely by fibrils that are shorter, more highly twisted, or based on a different peptide fold than the fibrils in lesion A. Lesion (C) is dominated by a strong peak at a scattering angle that corresponds to a spacing of about 4.15 Å (*q* = 1.51 Å^–1^) while missing a 4.7 Å peak. This cannot arise from a cross-β structure. A number of lipidic structures exhibit scattering at about 4.1 Å. Since this “lesion” is immediately adjacent to a blood vessel, it could correspond to a lipid inclusion that was not washed out by tissue processing. X-ray scattering from lesion D has a distinctly different intensity distribution. Its position in the heat map corresponds to a lesion that stains for tau in a serial section and appears to be devoid of staining for Aβ. Thus, it is highly likely that the scattering from lesion D is due to tau.

**FIGURE 5:**
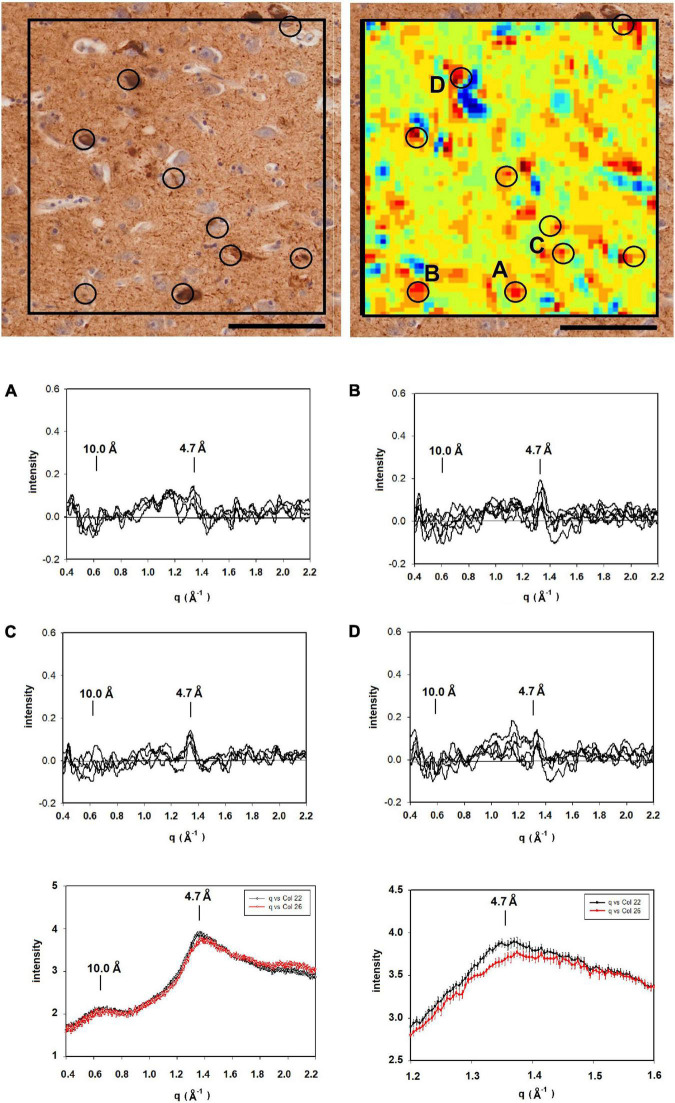
(Left) Serial section of entorhinal cortex of a Down Syndrome case with a secondary diagnosis of Alzheimer’s disease (ADNC) stained for tau (left) compared to a map of the intensity of 4.7 Å scattering from an adjacent unstained section (right). Immunohistochemistry indicated that Aβ was absent from these sections. **(A–D)** Scattering in the wide-angle region from lesions **(A–D)** in the corresponding x-ray scattering map (top right) indicated that the scattering was most intense at 4.7 Å, indicating high concentration of cross-β structures. (bottom) Plots of the average and standard deviations of lesions (black) and tissue (red) in the section shown at the top. Greatest difference between lesion and tissue is in the regions around 4.7 Å spacing. Scale bar 100 μ.

Figure 5 is derived from samples taken from the entorhinal cortex of a Down Syndrome case with a secondary diagnosis of Alzheimer’s disease (ADNC) with tau and Aβ pathology consistent with Braak Stage VI and Thal stage 5. As is typical for the entorhinal cortex, there is extensive tau pathology and little to no Aβ observed in this brain region. The tau-stained section (left) exhibited numerous examples of tau pathology including neurofibrillary tangles; neuritic plaques and dystrophic neurites. Numerous lesions as observed by XMD correspond to regions of tau pathology in the tau-stained serial section in Figure 5. Strongest scattering appeared to correspond to locations of neurofibrillary tangles. Detailed examination of scattering from these lesions indicated that in most cases the strongest wide-angle scattering was at q ∼ 1.34 Å^–1^, corresponding to the 4.7 Å cross-β scattering peak. In some cases, the strongest scattering appeared to be shifted, suggesting either a non-canonical structure or re-arrangement of other constituents of the lesion. The expected scattering at 10 Å spacing (q ∼ 0.6 Å^–1^) is not observed. In most of these patterns, the scattering is stronger at q ∼ 0.4–0.5 Å^–1^ than at 0.6 Å^–1^. This appears to be a common feature of scattering from cross-β structures composed of tau, as seen in the computed patterns in Figure 2. By contrast, strong scattering at q ∼ 0.6 Å^–1^ in the absence of scattering at q ∼ 0.5 Å^–1^ is predicted for many Aβ structures that have been solved by cryoEM (data not shown). Thus, the scattered intensity from the lesions in Figure 5 is consistent with that expected from fibrillar tau.

**FIGURE 6:**
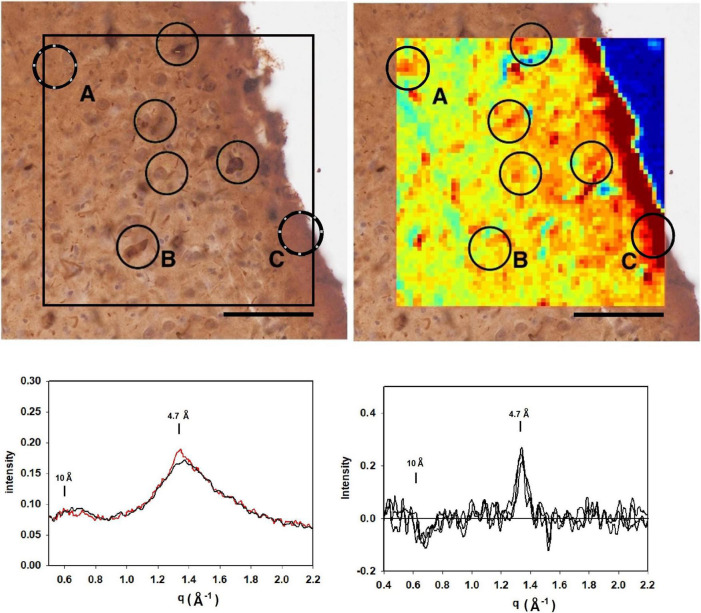
**(top left)** Image of a section of the entorhinal cortex of a subject with frontotemporal lobar degeneration with tau pathology (FTLD-tau) stained for tau. Comparison with the mapping of x-ray intensities **(top right)** identified numerous features common to both images. Analysis of the scattering patterns from these features indicated that most exhibited weak enhancement perhaps due to non-fibrillar tau (Figure 5) although most were much weaker **(bottom right)**. Feature exhibited a sharp 4.7 Å feature indicative of the presence of a cross-β structure **(bottom left)**. Given the complete absence of Aβ in this region (by immunostaining), this feature is interpreted as arising from fibrillar tau structures.

Figure 6 is analysis of a section from the entorhinal cortex, stained for tau from a subject with frontotemporal lobar degeneration with tau pathology (FTLD-tau) with MAPT mutation P301L (Braak stage IV; Thal stage 2) and no indication of Aβ deposits by neuropathology. Mapping 4.7 Å intensity across this ROI identified numerous potential lesions exhibiting strong scatter. The immediate vicinity of the tissue edge exhibited diffuse intensity at 4.7 Å that was significantly above average, perhaps reflecting a thickening of the tissue as it pulled away from the mica during drying. The tissue edge acted as a fiducial mark for putting the heat map and immunostained image in register. As seen in other images, some lesions in the heat map align well with features in the immunostained serial section, whereas others do not. This may be due to the presence of lesions that do not extend across both of the 20 μ thick sections or to staining of non-fibrillar tau. Examination of the scattering patterns from the more prominent features of the heat map indicate at least two classes of pattern—one, observed infrequently—exhibited a sharp reflection at 4.7 Å spacing and the other, more common class that exhibited a broader distribution of difference intensity. The sharp intensity peaks at 4.7 Å spacing are consistent with the presence of fibrillar structures made up of a cross-β structure. The complete absence of Aβ pathology combined with the high level of tau staining in these sections strongly suggests that this scattering is coming from fibrillar tau, but we cannot exclude the possibility that it is due to Aβ. Patterns with a broad intensity difference were more common and included much of the tissue margin that stained strongly for tau. The difference between these scattering patterns and patterns from regions of tissue judged to be devoid of lesions was too weak to unambiguously interpret in terms of the constituent structures.

## Discussion

The results presented here demonstrate that XMD, when utilized in coordination with neuropathological examination—and immunostaining in particular—can provide a wealth of information about the distribution of fibrillar polymorphs in human brain tissue. Ultimately, we would like to use this approach to address questions about the molecular mechanisms of disease progression. In order to do that, it will be necessary to collect the kinds of data presented here across multiple subject populations, at different stages of disease and multiple regions of the brain. The human brain is heterogeneous at all relevant length scales, providing a challenge for experimental design and data interpretation. Nevertheless, the data presented here provide some measure of the kinds of information that can be derived from XMD studies. Some trends noted in these preliminary studies deserve mention.

Interpretation of the scattering patterns that were observed from lesions in a single 300 × 400 μ XMD scan of tissue from a subject with a presenilin mutation (Figure 3) was made possible largely through the correspondence of XMD scans with immunohistochemistry. Some lesions observed by XMD corresponded to those seen in sections stained for tau; others in sections stained for Aβ. The correspondence made it possible to distinguish between scattering from Aβ and from tau. Furthermore, the unambiguous identification of lesions as due to Aβ make it possible to identify lesions in which the Aβ fibrils take on distinct structures. Differences observed among the scattering from lesions composed of Aβ peptides were of a magnitude and nature that could be due to differences in the twist of fibrils; in the distribution of fibrillar lengths or the peptide fold making up the core of the cross-β structure. Scattering from different parts of the same lesion exhibited very little variation except in overall intensity (which is proportional to the abundance of fibrils within different scattering volumes). The one exception apparent in the data reported here (lesion B in Figure 3) may suggest that some diversity within lesions is possible. XMD data that differed significantly from that expected of cross-β structures was also observed. For instance, “lesion” C in Figure 3 exhibits a sharp 4.15 Å peak indicating that it is likely composed of different molecular constituents—in this case, most likely lipidic in nature.

Variation in the scattering from different lesions composed of fibrillar tau was apparent through comparison of patterns in Figures 3, 5, 6. For the most part, the cross-β scattering patterns in lesions examined in the Down syndrome case (Figure 5) were very similar to one another although some variation was observed in the height and shape of the 4.7 Å cross-β peak and in the intensities in the ∼ 15 Å region. However, comparison of scattering from lesions in the Down syndrome case (Figure 5) with the MAPT mutation case (Figure 6) indicates significant differences in the structure of the cross-β core. The tau structures in these two cases must differ in significant ways. The 4.7 Å scattering from the tau lesion in the familial case (Figure 3 lesion “E”) is also distinct, not prototypical of cross-β structure. Although it is similar to scattering patterns predicted for some of the high-resolution tau structures observed by cryoEM (data not shown), it is possible that this lesion contains non-fibrillar tau or that other constituents are contributing to the observed scattering. The significance of this variation will become clearer with collection of additional data from other tissues and more extensive modeling of the scattering patterns predicted from high resolution cryoEM structures.

Differences in scattering among lesions composed of Aβ (or of tau) appear adequate to distinguish among structural polymorphs. The differences extend from the small angle region to beyond the 4.7 Å reflection and may be due to differences in fibril structure or to variations in their environment. Alterations in fibril structure that may alter observed scattering include the twist of fibrils; the distribution of fibrillar lengths or the peptide fold making up the core of the cross-β structure. Distinguishing among these possibilities should be possible through model building on the basis of the many high-resolution cross-β structures now available. The distinction is important. Changes in twist of a fibril involve low-energy transitions and can often be observed in a single electron microscope image. They may reflect innocuous variations that arise due to small differences in microenvironment. Differences in the fold of a peptide, however, are more likely to arise through independent nucleation events and reflect differences in the “strain” of a fibril that may have clinical relevance.

Changes in twist can alter the position of the 4.7 Å peak, with greater twist expected to shift the peak to higher scattering angle. This shift has been observed (Liu et al., 2016) and interpreted in terms of the “doublet” of intensity reported in the 4.7 Å region of scattering patterns from many amyloids (Sunde et al., 1997). Most of the patterns we have observed *in situ* appear to have a single peak at ∼ 4.7 Å spacing, but there is significant variation in the degree to which they have shoulders at higher angles that may be a reflection of a “doublet” structure or extended intensity at lower angles. These variations are landmarks of structural polymorphism that should become increasingly interpretable as the ensemble of patterns that we collect is expanded.

These studies have only begun to probe the diversity of information accessible using x-ray microdiffraction. The correlation of XMD with neuropathological examination of serial sections generates information not accessible to either method alone. Correlation of a broader range of samples including different regions of the brain in a single individual and different stages of disease in a specific subject population should provide significant additional insights into mechanisms of disease progression. Mapping the distribution of polymorphs within and among lesions will provide a measure of the degree to which disease progression is driven by prion-like spreading vs. independent nucleation. Prion-like migration should spread identical fibrillar structures throughout the tissue; whereas independent nucleation events could generate a variety of structures. Given the sensitivity of both Aβ and tau nucleation to their microenvironments, independent nucleation events, should they occur, might be expected to generate a broad range of distinct structures within the diverse microenvironments of the brain.

## Data Availability Statement

The raw data supporting the conclusions of this article will be made available by the authors, without undue reservation.

## Ethics Statement

Ethical review and approval were not required for the study on human participants in accordance with the local legislation and institutional requirements. Written informed consent for participation was not required for this study in accordance with the National Legislation and the Institutional requirements.

## Author Contributions

AB carried out the majority of data analysis in collaboration with PN. TC prepared all samples and took all optical micrographs. DO provided insights into the neuropathology of subjects. BH provided overall guidance for the project, detailed comments on neuropathology, and manuscript. LY collected all XMD data and provided extensive advice on data analysis. LM conceived the project, provided overall guidance for the work, and wrote most of the manuscript. All authors contributed to the article and approved the submitted version.

## Conflict of Interest

The authors declare that the research was conducted in the absence of any commercial or financial relationships that could be construed as a potential conflict of interest.

## Publisher’s Note

All claims expressed in this article are solely those of the authors and do not necessarily represent those of their affiliated organizations, or those of the publisher, the editors and the reviewers. Any product that may be evaluated in this article, or claim that may be made by its manufacturer, is not guaranteed or endorsed by the publisher.
